# Amigos de Fibro (Fibro Friends): development of an educational program for the health promotion of fibromyalgia patients

**DOI:** 10.1017/S1463423621000773

**Published:** 2022-08-04

**Authors:** Mateus Dias Antunes, Ana Carolina Basso Schmitt, Amélia Pasqual Marques

**Affiliations:** Department of Physiotherapy, Speech-Language Pathology and Audiology, and Occupational Therapy, Faculty of Medicine, University of São Paulo, São Paulo, SP, Brazil

**Keywords:** Fibromyalgia, health education, health promotion, patient-centered care

## Abstract

**Background:**

Educational practices are indicated to promote the health of people with fibromyalgia in primary health care. We aimed to develop an educative interdisciplinary program intended at the health promotion of individuals with fibromyalgia.

**Methods:**

It is a study protocol that was developed following three phases in the city of São Paulo city, Brazil. Qualitative research was carried out, through a focal group, with 12 individuals with fibromyalgia and 10 health professionals. A thematic content analysis was made according to the content proposed by Bardin.

**Results:**

Fibro Friends is an interdisciplinary program with educational approaches that must be performed in 15 meetings, once a week for 1 h and 20 min. Participants were the following professionals: a Physiotherapist, a Doctor, a Psychologist, a Nutritionist, a Nurse, a Pharmacist/Druggist, a Speech Therapist, an Occupational Therapist, a Naturopath, and a Social Worker. A physical exercise program will also be carried out. The professionals must discuss in a lecture, conversation hearing, and/or group dynamic, about strategies to promote health and pain control in fibromyalgia.

**Conclusion:**

Fibro Friends is a program presenting educational interdisciplinary information to individuals with fibromyalgia, being considered a trend to future care. Fibro Friends is a practical guide, logical, and efficient to patients with fibromyalgia at the basic attention to health.

## Introduction

Fibromyalgia is a complex multifactorial and of unknown etiopathogenesis, characterized by spread musculoskeletal pain and specific painful points, which are specific to palpation (tender points) and, frequently, associated to tiredness, somnolence, somatic, and cognitive symptoms with psychical disturbances (Wolfe *et al.*, 2016). The total prevalence of fibromyalgia in the general population ranges from 0.2% to 6.6% and is most frequent in women (Marques *et al.*, 2017). The fibromyalgia burden is considerable, with psychosocial consequences, as well as in the basic and instrumental activities of daily life (Antunes *et al.*, 2016; Schaefer *et al.*, 2016; Fitzcharles *et al.*, 2018). Beyond that, fibromyalgia’s economic costs are high and are considered a public health problem (Cabo-Meseguer *et al.*, 2017). The ideal treatment for fibromyalgia requires an interdisciplinary approach, including the association of pharmacological and nonpharmacological therapy (Braz *et al.*, 2011). The pharmacological treatment is essential and performed individually, remaining as a common element in most fibromyalgia cases, while the nonpharmacological treatments are centered on the adaptation process to face the fibromyalgia symptoms (Oliveira and Almeida, 2018).

The recommendations of the European League Against Rheumatism to Fibromyalgia management indicates that the most important strategy must be on the patient’s education. Health education is one of the main items to enable the health promotion at primary health care (Carneiro *et al.,* 2012), and must prepare each individual to take control and responsibility for their health (Nutbeam, 2018; Oliveira *et al.*, 2019). Studies carried out worldwide are still data representations of disciplinary educational interventions, without interdisciplinarity as the focus of attention (Antunes *et al*., 2021; García-Ríos *et al.*, 2019). In this sense, the study objective was to develop an educational interdisciplinary program aimed at the health promotion of individuals with fibromyalgia. This study intends to bring the patient’s and the health professional’s perspectives to the design, building, and implementation of an interdisciplinary intervention aimed at improving the health and transforming the healthcare services in Brazil.

## Methods

It is a study protocol with a qualitative approach. It was carried out in a Basic Health Unit in the city of São Paulo city, São Paulo State, Brazil. The study was approved by the Research Ethics Committee of the Medical Faculty of the University of São Paulo, approval number 3.197.778.

Participants received written and verbal clarification about their participation in the study. The individuals with fibromyalgia and the health professionals received and signed an informed consent form. Fibro Friends is based on integrative community therapy which is a guide to health practices. The integrative community therapy is a therapeutic model created after World War II, when an American psychosociologist, Kurt Lewin, worked through a group of people with therapeutic results. He asserts that individuals feel a therapeutic benefit when listening to people who share their problems, as they talk about how these problems can be solved. The integrative community therapy was developed in Brazil to create supportive social networks aimed at life promotion, raising resources, and the individuals, families, and communities’ competencies, to promote empowerment. This practice is interesting for the promotion of wellbeing, health, and social rights, it seeks to enhance the group’s therapeutic dimension, to value the cultural background, as well as the knowledge gathered by the life experience of each individual and is enlisted in the “Integrative and Complementary Practices on Health in Brazil” (Práticas Integrativas e Complementares em Saúde no Brasil) (Barreto, 2008). Fibro Friends Program presents as a goal, to guide and teach self-care techniques to individuals with fibromyalgia, and to its construction, the following steps were implemented.

### First phase: requisite analysis, contents, and establishing the programs’ objectives

A total of 12 individuals with clinical fibromyalgia diagnosis were invited. The criteria were confirmed by an evaluator according to the ‘Classification Criteria of the American College of Rheumatology’, revised version of 2016 (Wolfe *et al.*, 2016), the participants were users of the Brazilian Unified Health System (SUS – *Sistema Único de Saúde*), with complete elementary school grades to participate in qualitative research through group discussion. Since it is a qualitative approach, the sample is defined by the saturation of the question to be analyzed. There was not, therefore, a previous statistic estimate to define the number of subjects to compose the sample. Therefore, once we have deep and sufficient information for a scientific investigation, data collection might be ended and considered finished. The interview technique, due to the time involved and deepness of the search, limits the number of individuals to be researched. The most common and indicated group technique in qualitative researches is the focal group. The focal group is a type of interview which is generally carried out with a small and homogeneous group from 6 to 12 people (Pelicioni, 2001). The group meeting aimed at identifying, through these individuals’ speeches, the needs, and problems reported by the individuals with fibromyalgia and the possibilities for an interdisciplinary professional team to meet these requirements and to solve the problems.

Also, it was requested by the responsible physiotherapist, that they specified the number, the time length, and the frequency of meetings. At the end of this phase, the two physiotherapists responsible for the project, have listed the topics mentioned by the individuals suffering from fibromyalgia. Moreover, 10 professionals dealing with primary health care at the Unified Health System (SUS) were invited to participate in the group discussions about the program development. The choice of the number of professionals was according to the interdisciplinarity and with the possibility of a positive contribution to the aspects related to the quality of life of an individual with fibromyalgia. The invited health professionals were a Physiotherapist, a Doctor, a Psychologist, a Nutritionist, a Nurse, a Pharmacist/Druggist, a Speech Therapist, an Occupational Therapist, a Naturopath, and a Social Worker. This meeting aimed at questioning the professionals about what interdisciplinary matters are important, practical, and necessary to be approached by the program in primary health care, based on the professionals’ experience and on the current scientific evidence to fibromyalgia treatment, as well as the number, the time length, and the frequency of meetings. The project proposes to do group work rather than individual treatments. The program’s proposal is an interdisciplinary approach and all professionals that are in primary health care could participate.

### Data collection

The qualitative research was carried out through spontaneous oral speeches, in which the individuals with fibromyalgia and the healthcare professionals have spontaneously spoken about the investigated theme. Qualitative research considers exclusively meanings and processes, and not measurements. The results are presented descriptively and not numerically (Salmon and Young, 2018). It is important to highlight that, this type of research depends on the precision of the interviewer’s intuition and ability in dealing with resources and techniques to represent the phenomenon, as there are not formulated hypothesis, and also, there are no absolute criteria for data collection (Canzonieri *et al.*, 2009). In this study, the saturation criterion was not applied, because the purpose was to collect responses from professionals and patients who were invited to participate in the study. The questioning of the professional and the patient were executed the same way, allowing them to freely express themselves. In all the conversations, interviewers and interviewees talked in a calm voice tone and a tranquil verbal and body expression. There were no difficulties. The interview was performed using a portable digital recorder (model Zoom H4N PRO – 2018).

Data collection took place at the Basic Health Unit, in a private location, through group interviews combined with participative observation techniques, from September 3rd, 2019 to October 22nd, 2019, at days and hours previously established by the interviewer. The average data collection time with each group was 1 h and 30 min. The script elaborated by the researchers had pertinent questions to the researched problem. The average data collection time with each group was 1 h and 30 min. The orienting script, elaborated by the researchers, has contemplated pertinent questions to the researched problem.

### Content analysis

For the content analysis, the data collected were transcribed, organized with Excel and Word software, and the answers were mapped, allowing a panoramic content reading. Afterward, these data were analyzed through content analysis proposed by Bardin (1979), specifically the thematic content analysis (Minayo, 2008), which has allowed the organization of textual content, creating categorizations to enable inferences and to recognize patterns.

The different phases of content analysis have been organized around three poles:

1. Pre-analysis (audio hearing and checking the notes)
2. Material exploration (interview transcription)
3. Data treatment (inference and Interpretation)

In this phase, a tool was used for the visualization and mapping of the most recurrent topics in all interviews analyzed. The speeches were categorized by items in a table which considered those that refer to facing the problem, interdisciplinary information, and educational program structure. These categories were created based on Werneck *et al.* (2009) who attribute the main criteria to the creation of healthcare protocols and organization of health services.

### Second phase: program development

The program development was performed through information supplied by individuals with fibromyalgia and also by healthcare professionals, a program structure presenting in each thematic the conditions offered by the professional, the activities to be performed by the patients and the activity product, as well as the number, the time length, and the frequency of meetings. This phase was carried out by two responsible physiotherapists. The conditions refer to the main theme and the way to approach it. Concerning the activities to be carried out by the participants, in this item, there is a detailed account of which is expected that people with fibromyalgia learn and make in each meeting. The participants’ activity product refers to the knowledge that the participant of the Fibro Friend Program must have after each meeting. Those items of program creation were based on Brasil (2007).

### Third phase: program validation

In this phase, after the program development, the two physiotherapists responsible for the project have presented the program structure to the 12 individuals with fibromyalgia who have participated in the first phase of the study in a group meeting. It was also realized a meeting in a group with the 10 professionals who participated in the first phase of the study to do content validation. The two meetings (patient group and professional group) were carried out on different days and hours, and the posterior meeting of patients and professionals was not carried out. This validation step sought to allow individuals with fibromyalgia and professionals, getting to know the program structure and the possibility of adjusting before starting the viability study phase.

## Results

The results will be presented in the thematic category description defined by the professionals and individuals with fibromyalgia. The interdisciplinary program structure of promotion to fibromyalgia patients was conducted using a combination of activities that have followed the series of themes addressed which are based on the National Policy of Health Promotion. As a primary outcome, the study participants have chosen the ‘quality of life’, the secondary ones were: ‘level of pain’, ‘sleep quality’, and ‘self-care management’. A physical exercise program will also be carried out. To an educational program in health, directed to people with fibromyalgia, 15 weekly meetings lasting 1 h and 20 min are suggested on Tuesdays afternoon (Table 1).

**Table 1:** Description of Fibro Friends

Meeting	Theme	Professional	Conditions offered by the professional	Activities performed by the participant	Products of participants’ activity
1	Presentation of the program and socialization	Physiotherapist	The physiotherapist coordinating the program should present the interdisciplinary health promotion program for people with fibromyalgia. Also, the physiotherapist must socialize the participants by introducing them to each other. In this meeting, the responsible physiotherapist must present the schedule (dates, time, frequency, period of the day, and day of the week) and will be able to answer the questions of individuals with fibromyalgia about the way the program works	Participants must know what the program’s proposals are, the schedule and the themes they will get to know throughout the program. They should introduce themselves	Know the program activities and socialize the participants
2	Knowing fibromyalgia	Doctor	The doctor should discuss in a lecture, conversation circle, and/or dynamics about the concept of fibromyalgia, the associated conditions, risk factors, classification criteria, living with symptoms and pharmacological, and nonpharmacological and strategies In this meeting, the professional should also advise on some self-care measures to promote their health and demonstrate them. The professional should ask the participants what their questions and preconceptions about fibromyalgia are and answer them	Participants should learn the true concepts of fibromyalgia, the causes, risk factors, tender points, symptoms, living with symptoms, non-pharmacological strategies used by the group to treat their symptoms Moreover, participants should demonstrate self-care practices that will be passed on by the doctor to promote their health Participants will be able to ask the doctor questions regarding fibromyalgia	Know about the concepts of fibromyalgia and other aspects that interfere with the quality of life, as well as some ways of self-care
3	Health production and care	Nurse	The nurse should discuss in a lecture, conversation circle, and/or dynamics about the importance and incorporation of self-care in daily practice The nurse must also address humanized care practices, supporting the needs of fibromyalgia individuals, to strengthen their participation in society Moreover, the nurse must discuss the opportunities for coexistence, solidarity, respect for life and strengthening bonds in fibromyalgia	Participants should reflect on the importance of self-care to promote their health. They will also carry out some care practices according to their needs so that they can play actively in society. Participants must know the living environments that produce health and promote self-care	Understand about practices and environments that favor self-care in fibromyalgia
4	Family and work	Social worker	The social worker should discuss in a lecture, conversation circle, and/or dynamics about the relationship between fibromyalgia symptoms and work difficulties The social worker must help the participant to understand the importance of family support in fibromyalgia Also, must demonstrate strategies that promote health in the work and family environment	Participants must identify the importance of work to improve the negative conditions of fibromyalgia Participants must understand the role of their family in the pursuit of quality of life. They need to perform some of the strategies provided by the professional to improve the relationship of fibromyalgia with family and work	Understand the importance of family support and practices to promote health at work
5	Body practices and physical activity	Physiotherapist	The physiotherapist should discuss in a lecture, conversation circle, and/or dynamics about the main body practices and physical activity and its benefits in fibromyalgia. The appropriate type of exercise for fibromyalgia should also be important He/she must demonstrate and teach physical exercises that promote health in fibromyalgia Show the public spaces that have bodily practices and physical activity and the correct way to use them. Also, inform that the individual may no longer have fibromyalgia one day	Participants must understand the importance of body practices and physical activity in improving the clinical condition of fibromyalgia Participants must perform the body practices and physical activity that will be taught by the physiotherapist to seek a quality of life	Know and experience the main body practices and physical activity that promote health in fibromyalgia
6	Healthy and adequate feeding	Nutritionist	The nutritionist should discuss in a lecture, conversation circle, and/or dynamics about what food is adequate and healthy. Present the main healthy and adequate foods. He/she must demonstrate how to prepare healthy foods that promote food and nutritional safety Offer practical and easy recipes that support healthy food promotion	Participants must understand the importance of healthy and adequate food to promote health in fibromyalgia Participants should observe the guidelines given by the nutritionist on healthy and adequate recipes and foods	Know and experience the main actions that promote adequate and healthy eating
7	Health and Well-being	Psychologist	The psychologist should discuss in a lecture, conversation circle, and/or dynamics about the implication of fibromyalgia in mental health Also, he/she must address the anxiety and depression issues and ways of coping with them The psychologist must demonstrate strategies that seek to cope with mental illness	Participants must understand the importance of controlling psychic symptoms in fibromyalgia. Participants should also know about the interrelationship of fibromyalgia with anxiety and depression. Participants must carry out the strategies and dynamics proposed by the psychologist to preserve mental health	Understand about the relationship between psychological issues and fibromyalgia and be encouraged to face mental illnesses
8	Pharmacologic approach	Pharmacist/druggist	The pharmacist should discuss in a lecture, conversation circle, and/or dynamics about the main drugs with mechanisms involved in inhibiting pain and other fibromyalgia symptoms. Present possible side effects He/she must inform on the importance and the ways of management and medication adherence The pharmacist must demonstrate the importance of combining pharmacological and nonpharmacological methods	Participants must understand the importance of pharmacological treatment for the control of fibromyalgia symptoms. Participants must know how to manage and adhere to the use of medicines	Understand the importance of pharmacological treatment
9	Activity integration	Physiotherapist	The physiotherapist responsible for coordinating the project should inform the participant about the week when there will be no face-to-face meeting. The professional should encourage the participant to seek the interaction of the strategies individually	Participants must work independently on the contents of previous meetings	Assimilate the topics covered in previous meetings
10	Integrative and complementary practices	Naturopath	The naturopath should discuss in a lecture, conversation circle, and/or dynamics about understanding the main integrative and complementary practices, exploring their health benefits Demonstrate and/or perform some integrative and complementary practices for the participant to do it at home. Furthermore, he/she should encourage the practice of these activities in the participants’ daily lives Valuing the participants’ popular and traditional knowledge and integrative and complementary practices	Participants must know the benefits of integrative and complementary practices in their health. Perform the demonstrations of some complementary and integrative practices that will be passed on by the naturopath. They must explain the knowledge of popular and traditional knowledge of methods that promote health. Participants should feel encouraged to include integrative and complementary practices in their daily lives	Know and incorporate integrative and complementary practices in daily routine for health promotion
11	Activity integration	Physiotherapist	Same activities as meeting 9	Same activities as meeting 9	Same activities as meeting 9
12	Occupational performance	Occupational therapist	The occupational therapist should discuss in a lecture, conversation circle, and/or dynamics about the importance of occupational therapy in the control of fibromyalgia symptoms He/she should inform the experience and difficulties in carrying out basic and instrumental activities of daily living in people with fibromyalgia and the possibilities of improvement with adaptations Also, the occupational therapist should address functional adaptations to improve the quality of life. Offer the equipment that helps individuals with fibromyalgia in the performance of basic and instrumental activities of daily living	Participants must know the importance and benefits of occupational therapist intervention in their daily routine. They must perform demonstrations of some practices that will be presented by the occupational therapist. Participants should feel encouraged to include the adaptations that were informed by the professional to their daily routine	Encourage the participant to make known adaptations in their daily routine to promote changes in functionality and quality of life
13	Activity integration	Physiotherapist	Same activities as meetings 9 and 11	Same activities as Meetings 9 and 11	Same activities as Meetings 9 and 11
14	Sleep quality	Speech therapist	The speech therapist should explain in a lecture, conversation circle, and/or dynamics about the importance of speech therapy in the control of fibromyalgia symptoms and present the main sleep disorders. The speech therapist must show and encourage the use of relaxation and breathing techniques for symptom relief, body perception, and sleep quality	Participants must know the benefits of the techniques to improve sleep quality. They should reproduce the demonstrations of some practices that will be shown by the speech therapist. Participants should feel encouraged to include practices that help with impaired sleep related to general pain	Encourage the participant to use the techniques to improve sleep quality
15	Retrospective	Physiotherapist	The physiotherapist responsible for coordinating the project should discuss, in a lecture, conversation circle, and/or dynamics, motivation strategies so that the participants continue to practice the activities they learned in the program through the initial retrospective	Participants must be encouraged to keep practicing the activities they learned during the program, adopting them in their lives. Also, participants can disseminate this knowledge to their families and acquaintances	Adopt the knowledge and practices they acquired throughout the program for a lifetime

The schedule summary of Fibro Friend is described in Table 1.

## Discussion

An interdisciplinary health promotion program was jointly created for the health promotion of individuals with fibromyalgia, named Fibro Friends’. The Patient’s education is considered as the first step for the self-management of symptoms of fibromyalgia (García-Ríos *et al.*, 2019). A patient’s education is defined as any set of educational activities planned by qualified professionals and intended to improve health behavior and/or the patient’s health. Beyond that, the patient’s education seeks to inform and restructure the perceptions about the disease (García-Ríos *et al.*, 2019). To reduce the disparity between the perception of pain that the patient and the health professional have and their treatment, it is necessary to change the maladaptive perception that patients have of the disease, reconceptualizing the pain, the disease itself, and the symptoms presented (Nijs *et al.*, 2011; García-Ríos *et al.*, 2019).

Therefore, the patient’s education must be based on approaches that provide knowledge and information about the disease, the planned treatment, the strategies, and the expected outcomes (Hawkins, 2013; Häuser *et al.*, 2017). To develop a treatment that meets the patients’ needs, obtaining a long-term adhesion, it is essential to understand the patients’ perspectives – their expectations, their living experience of intervention, their acceptability, their lifestyle adequacy and results, and efficacy on the perceptions (Ashe *et al.*, 2017).

The primary management must involve the patient’s education and the focus on nonpharmacologic therapies. In the case of nonresponsive treatment, other therapies must be adapted to the specific individual’s needs and might involve psychological therapies, pharmacotherapy, and/or a combined rehabilitation program (Macfarlane *et al.*, 2016). To deal with the chronic pain condition, cognitive behavioral therapy is indicated to allow daily life activities and professional and social life participation (Sauer *et al.*, 2011; Gregório *et al.*, 2018).

In this sense, this program will be based on cognitive behavioral therapy which is some of the therapies focused on behavior, emotions, and symptoms. This approach has an emphasis on the individual’s current situation rather than in past situations. Thoughts may represent cognitive alterations which might influence directly the individual’s health condition. In this sense, this therapy seeks to change the individual’s behavioral aspects, stimulating him/her to review inadequate attitudes and beliefs which might be negatively influencing their health condition (Bennett and Nelson, 2006; Williams, 2006; Minelli and Vaona, 2012).

Qualitative studies about the fibromyalgia patient’s perspectives highlight worries, as the lack of information provided by healthcare professionals; a general lack of understanding by the family, friends, and society; trouble in adapting to fibromyalgia’s pain, fatigue, and powerlessness (Ashe *et al.*, 2017). In this sense, it is evident that the need to continue studying the therapeutic approaches to fibromyalgia worldwide, mainly in Brazil (Cabo-Meseguer *et al.*, 2017).

In this sense, the creation of combined programs is encouraged and, in particular, in the follow-up, the habit modification and self-efficacy, in response to some of the current limitations in the intervention with these patients (Pérez-Velasco and Peñacoba-Puente, 2015). Thus, the ‘Fibro Friends Program’ will have as a contribution to the health system, qualified information which will serve as a support to healguide the effectiveness, evaluation, and viability of a health promotion program to people with fibromyalgia in Brazil.

## Conclusion

Fibro Friends is the first interdisciplinary program in Brazil to promote health for fibromyalgia individuals. It was created based on scientific evidence, clinical experience, and patient’s opinion. The authors suggest that healthcare professionals recommend using this program to their patients as an auxiliary resource to promote health in fibromyalgia.

### Practical implications

Fibro Friends was created from the joint action of patients and healthcare professionals. It can be an effective educational tool to be implemented at primary health care centers, promoting self-care, quality of life, and health promotion in individuals with fibromyalgia. Fibro Friends is an outstanding tool for patient education and counseling in Brazil.

### Future research

The next step is employing the ‘Fibro Friends Program’ through an important random clinical essay to assess the efficiency of the interdisciplinary program of health promotion in patients with fibromyalgia, and, soon, the researchers must provide new information about this subject. The protocol was registered at the Brazilian Registry of Clinical Essays (*Registro Brasileiro de Ensaios Clínicos* – ReBEC) under reference number: (RBR-3rh759).
